# A longitudinal study of quantitative pulmonary dynamic contrast enhanced MRI following COVID-19 infection

**DOI:** 10.1186/s12931-026-03582-w

**Published:** 2026-03-26

**Authors:** Adrienne E. Campbell-Washburn, Shreya Kanth, Matthew D. Thurston, Christine Mancini, Kendall J. O’Brien, Amanda Potersnak, Haiyan Wang, Julio Huapaya, David Regenold, Scott Baute, Ahsan Javed, Anthony F. Suffredini

**Affiliations:** 1 Cardiovascular Branch, Division of Intramural Research, National Heart, Lung, and Blood Institute, National Institutes of Health, 10 Center Drive, Building 10 Rm B1D219, Bethesda, MD 20892 United States; 2 Critical Care Medicine and Pulmonary Branch, Division of Intramural Research, National Heart, Lung, and Blood Institute, National Institutes of Health, 10 Center Dr, Bethesda, MD 20892 USA; 3 Critical Care Medicine Department, Clinical Center, National Institutes of Health, 10 Center Dr, Bethesda, MD 20892 USA

**Keywords:** SARS-CoV-2, COVID-19, MRI, Perfusion, Dynamic-contrast enhanced

## Abstract

**Background:**

Changes in pulmonary microvascular perfusion after SARS-CoV-2 infection remains poorly characterized due to limitations in longitudinal imaging. To provide a longitudinal evaluation of lung perfusion, we used quantitative dynamic contrast enhanced MRI (DCE-MRI) on a 0.55T MRI system in patients following COVID-19.

**Methods:**

In this prospective study, we performed quantitative DCE-MRI to generate perfusion maps on patients with a history of COVID-19 between June 2020 and June 2023. Imaging studies were divided into acute, recovery, convalescent, and extended study phases, collected over 3 years. Median lung perfusion, perfusion heterogeneity, perfusion defect percent, pulmonary transit time, arterial transit time, and transit time defect percent were measured. Perfusion metrics were correlated with pulmonary function tests (PFT), disease severity, cardiorespiratory symptoms, vaccine status, viral variant, and chest CT findings.

**Results:**

We included 84 post-COVID-19 patients and 156 separate DCE-MRI exams for analysis, and 10 healthy volunteers. Statistical significance between patients with COVID-19 and healthy volunteers was observed for perfusion defect percent in all study phases (*p* < 0.01). Five patients had visible perfusion defects. Lower median perfusion was found in patients with low diffusing capacity for carbon monoxide (DLCO) in the recovery phase (*p* = 0.02), and patients with heterogeneous perfusion maps in the acute phase were more likely to have low DLCO at their final PFT measurements (*p* = 0.01). During the convalescent phase, patients with residual symptoms had lower median perfusion (*p* = 0.01), unvaccinated patients had higher transit time defect percent (*p* = 0.0047), and ground glass opacities on CT were associated with lower median perfusion (*p* = 0.004).

**Conclusions:**

Pulmonary microvascular perfusion abnormalities are found months after COVID-19, with correlative findings in patients with persistence of pulmonary symptoms and impaired pulmonary function. Our findings further support the utilization of DCE-MRI in a broad range of pulmonary vascular disorders.

**Trial registration:**

clinicaltrials.gov NCT04401449 (registered 2020-05-22) and NCT03331380 (registered 2017-11-02).

**Supplementary Information:**

The online version contains supplementary material available at 10.1186/s12931-026-03582-w.

## Background

Vascular endothelial abnormalities and pulmonary vascular dysfunction are key features of several respiratory viruses and have been particularly noted with severe acute respiratory syndrome coronavirus 2 (SARS-CoV-2 or “COVID-19”) [1]. In the lungs, COVID-19 causes perfusion defects associated with microvascular damage, microthrombi, and pulmonary vasoconstriction [1–4]. Yet routine imaging of lung perfusion is often under-utilized in clinical practice and this information may change clinical management, particularly with pulmonary vascular pathologies. Dual energy CT and nuclear SPECT imaging have been explored for assessment of regional perfusion, but these methods require ionizing radiation [5]. Alternatively, low field (0.55T) cardiopulmonary MRI imaging is emerging as a valuable tool to assess lung microarchitecture, tissue characteristics, and quantitative regional perfusion mapping without ionizing radiation which makes it well suited for repeat serial imaging of patients [6, 7].

Dynamic contrast enhanced (DCE) MRI is a common method used to evaluate tissue perfusion by using a time series of image acquisitions during and following the injection of a gadolinium contrast agent, and has been applied in the lung previously [8]. DCE-MRI can be processed to provide 3D regional evaluation of pulmonary microvasculature. Two prior studies evaluated DCE-MRI in COVID-19 by semi-quantitative analysis of the time-to-peak contrast, wash-in, and wash-out rates in the lung parenchyma, using either pixel-wise maps or regional analysis [9, 10]. In both studies, differences between the post-COVID-19 group and healthy volunteers were identified.

DCE-MRI can be combined with pharmacokinetic modelling for full quantification of physiologically relevant parameters, including arterial plasma flow, volume of distribution of gadolinium, and vascular permeability. This approach requires quantitative modelling of the signal evolution curves relative to an arterial input function [11]. Model-based quantitative DCE-MRI has been applied in the lung previously [12–14], including to characterize pulmonary perfusion post-COVID-19 in a small number of patients [15].

Here we use repeated measures of quantitative DCE-MRI in a cohort of patients following COVID-19 infection, assess the evolution of pulmonary perfusion over time, and correlate perfusion measurements with clinical presentation and clinical metrics. We hypothesized that pulmonary perfusion abnormalities caused by microvascular damage would be detectable and may be related to associated clinical metrics.

## Materials and methods

### Study design

The COVID ARC-19 (Cardiopulmonary Inflammation and Multi-System Imaging During the Clinical Course of COVID-19 Infection in Asymptomatic and Symptomatic Persons, NCT04401449, registered 2020-05-22) is a natural history study designed to characterize the clinical and biologic effects of SARS-CoV-2 infection longitudinally. The study prospectively enrolled patients 18 years or older to the National Institutes of Health Clinical Center with COVID-19, confirmed by positive polymerase chain reaction. Patients were co-enrolled in a study for research heart and lung imaging using an investigational 0.55T MRI system (NCT03331380, registered 2017-11-02). Both studies were approved by the institutional review board at the NIH and all patients provided written consent for study participation. Some results of the COVID ARC-19 cohort have been previously published, with overlapping patient population. Specifically, plasma proteomics (90 patients) [16], longitudinal analysis of lung proteomics (45 patients) [17], CT analysis (45 patients) [18], and MRI images of lung structure (9 examples) [19–21]. This is the first publication of pulmonary perfusion results from the COVID ARC-19 patient cohort.

**Fig. 1:**
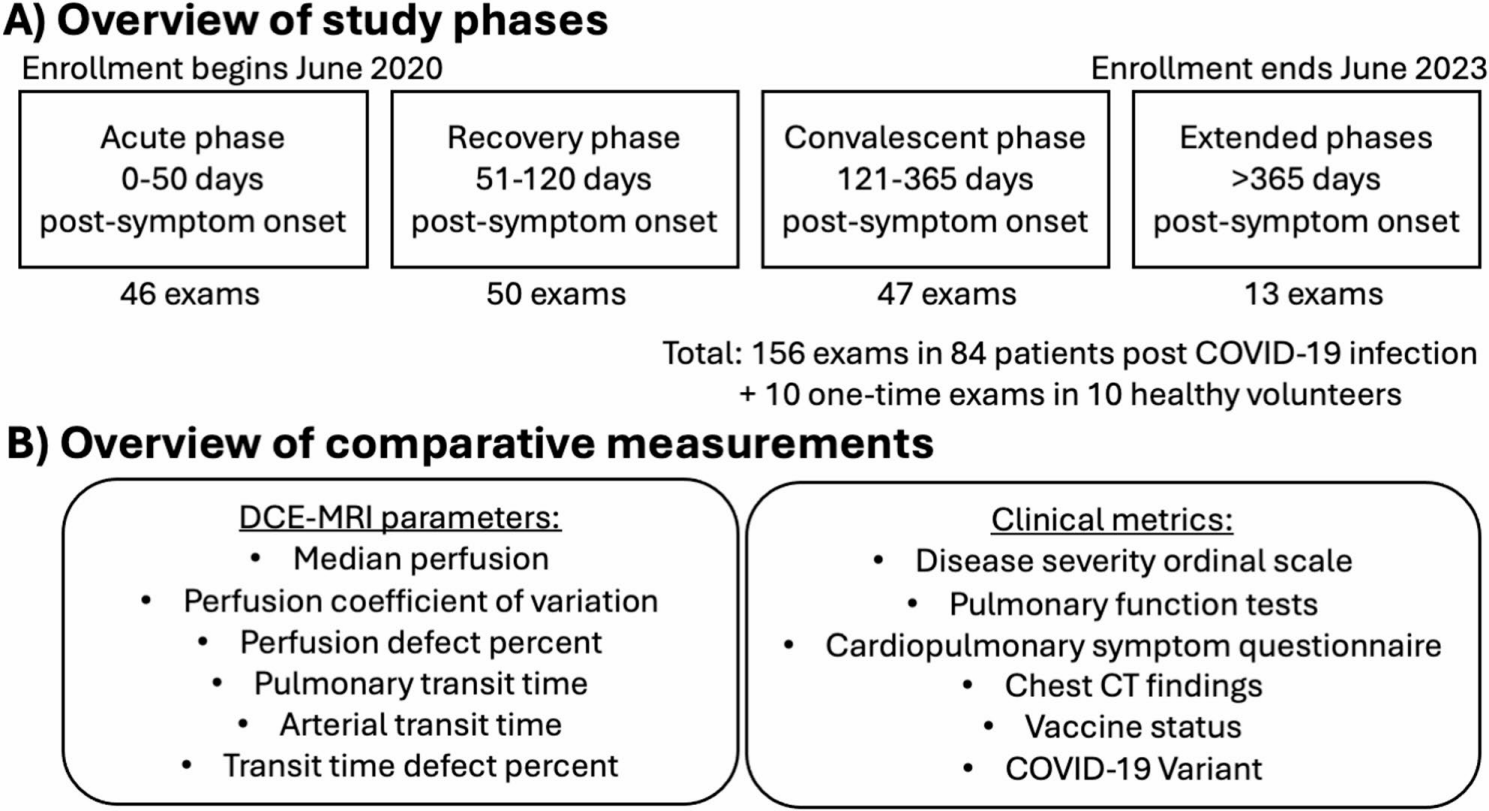
Summary of study design. **A** Overview of acute, recovery, convalescent, and extended study phases following COVID-19 infection. The number of exams performed in each phase are reported. **B** Overview of DCE-MRI quantitative perfusion metrics calculated from MRI data and the clinical metrics used for comparison

Our study design is summarized in Fig. 1. We performed longitudinal DCE-MRI imaging on patients with a history of COVID-19 between June 2020 and June 2023. Patients were imaged in 5 study phases post-symptom onset: acute (0–50 days post-symptom onset), recovery (51–120 days), convalescent (121–365 days), extended 1 (365–1000 days), extended 2 (> 1000 days). Normal values of quantitative perfusion parameters by DCE-MRI were determined from 10 healthy volunteers with no stated history of COVID-19.

### Imaging methods

We used a contemporary 0.55T MRI system (prototype MAGNETOM Aera, Siemens Healthcare, Erlangen, Germany) for imaging, which offers high-quality pulmonary images by virtue of reduced air-tissue susceptibility artifacts [22].

DCE-MRI used a dynamic 3D Cartesian gradient echo acquisition with a frame rate of 1.5s/volume (breath held, TE/TR/FA = 0.78ms/2.54ms/31°, FOV = 450 mm x 450 mm, resolution = 3.75 × 3.75 × 20mm^3^, 8 slices, see Supplemental Video 1 for example dynamic sequences). The total acquisition time was 37.5s, and participants were asked to hold their breath at end inhalation for as long as possible before slowly releasing it. Gadolinium contrast (Gadavist, Bayer) was injected (2 ml at 4 ml/s) to image the first pass of contrast through the pulmonary tissue.

### Image analysis

**Fig. 2:**
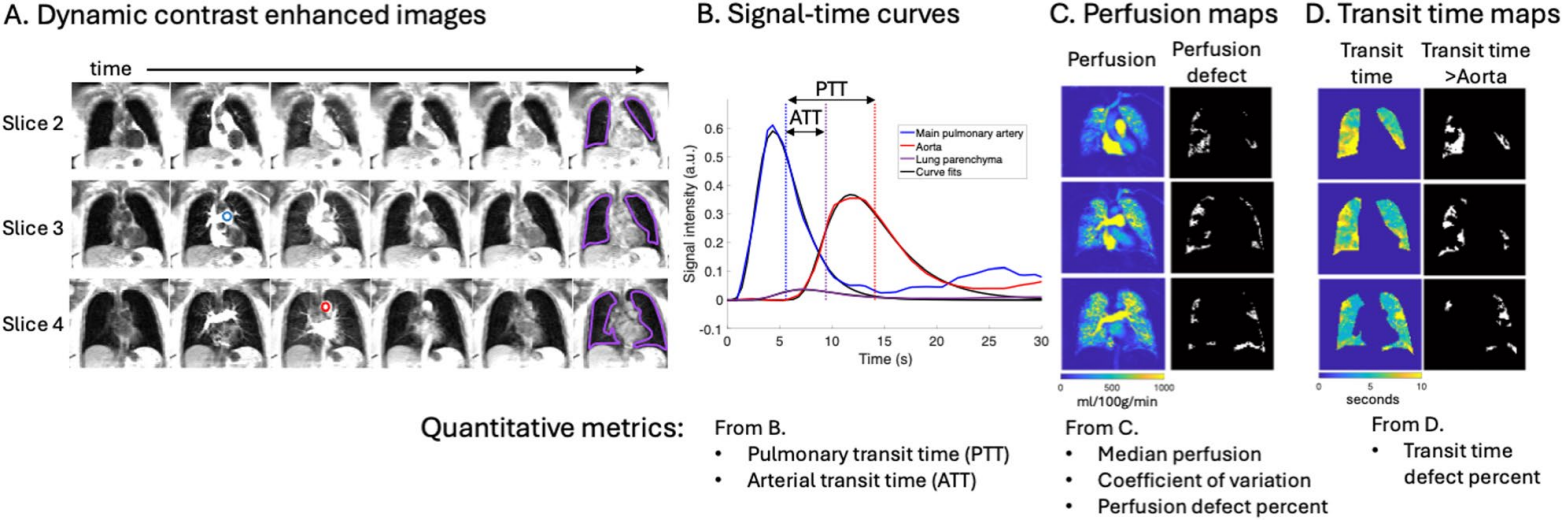
Quantitative pulmonary perfusion mapping by DCE MRI. **A** Example image timeseries in three slices is provided to illustrate the pass of gadolinium contrast bolus through the lung. The main pulmonary artery (blue circle), aorta (red circle), and lung (purple region) were segmented. **B** Signal-time curves are fit with a log-normal curve and curve centroids are compared to calculate the pulmonary transit time (PTT) and arterial transit time (ATT). **C** Pixel-wise quantitative perfusion maps are generated from the two-compartment exchange model using the main pulmonary artery signal as the arterial input function to plasma flow (“perfusion”) maps. Pixel-wise perfusion defect maps are generated to identify pixels with abnormally low perfusion. From perfusion maps, the median perfusion, perfusion coefficient of variation, and perfusion defect percent are calculated. **D** Pixel-wise transit time maps are generated by comparing the main pulmonary artery curve to pixel-by-pixel signal curves, and transit time defect maps are generated by identifying pixels where transit time is >PTT

Figure 2 provides example images and an illustration of the quantitative perfusion mapping pipeline. Tissue perfusion maps were calculated using pharmacokinetic modeling. We employed the two-compartment exchange model by using the mean signal from a region of interest in the main-pulmonary artery for the arterial input function. Perfusion modelling was performed using the open-source ROCKETSHIP software package [23] and all processing was performed in MATLAB R2022b (Mathworks, Natick, MA). Based on the two-compartment exchange model fit, the plasma flow parameter was used to measure pulmonary perfusion, and other parameters (e.g., blood volumes and permeability) were not used in our analysis. Lungs and main pulmonary artery were manually segmented by a single analyzer (ACW) drawing the regions-of-interest in image analysis

Several perfusion metrics were calculated from the perfusion map, as follows. Median lung perfusion was calculated from the central 6 slices, and the coefficient of variation (perfusion standard deviation/mean perfusion) was calculated as an assessment of perfusion heterogeneity. Regional perfusion defects were identified slice-by-slice by taking a threshold of < 40% of the slice-wise median perfusion and including only clusters of > 7 connected abnormal pixels. This thresholding approach was adapted from previously published methods used to calculate ventilation defect percent with hyperpolarized gas [24]. A perfusion defect map was calculated, as was the percent perfusion defect (defect volume / total lung volume x 100%).

Temporal contrast agent kinetics were also characterized. The pulmonary transit time (PTT) and arterial transit time (ATT) were calculated using the signal-time curves during contrast injection. Curves were fit to log-normal probability density function and the centroid of the fitted waveform was calculated [25]. PTT was estimated as the time between centroids for the main pulmonary artery and aorta signal-time curves, and ATT was estimated by the main pulmonary artery and mean lung signal-time curves. Additionally, pixel-wise transit time maps were computed for every pixel in the lung. Pixels having transit time > PTT were identified as “abnormal”, and a transit time defect map was generated by including only clusters of > 7 connected abnormal pixels.

The six perfusion parameters (median perfusion, perfusion coefficient of variation, perfusion defect percent, PTT, ATT, and transit time defect percent) were compared among study participants. Perfusion maps were also visually inspected for perfusion abnormalities.

### Comparisons to clinical metrics

Disease severity was assessed using a modified NIAID severity of illness ordinal scale (1 = mild, ambulatory; 2 = moderate, hospitalized with no oxygen or low-flow oxygen ≤ 6 L per minute nasal cannula; 3 = severe, hospitalized with high-flow oxygen > 6 L per minute on nonrebreather face mask or high-flow nasal cannula) [16, 26], and patients were grouped by their disease severity for perfusion analysis. Vaccination status and COVID-19 variant were recorded for all patients.

Pulmonary function tests (PFTs) were measured using spirometry and reported as percent predicted. Only measurements within 60 days of DCE-MRI were included. Fewer PFT measurements were available from the acute study phase, as our PFT lab was closed during peak pandemic to prevent aerosolization of SARS-CoV-2 virus. We evaluated the forced expiratory volume in one second (FEV1), the ratio of FEV1 to forced vital capacity (FEV1/FVC), and diffusing capacity for carbon monoxide (DLCO). DLCO is affected by changes in pulmonary blood flow or in the alveolar-capillary interface directly impacting the diffusion of CO. To evaluate this relationship, we grouped patients were grouped by normal and low DLCO (< 80% predicted) in each phase, as an indication of clinical lung function status. To assess the predictive value of perfusion measurements, the data were paired between acute phase perfusion and final study PFT measurement.

Patient histories and physical exams provided documentation of COVID-19 symptoms, and regular telephone questionnaires were completed throughout the COVID ARC-19 study with follow-up phone inquiries. Patients were grouped by a binary assessment of presence or absence of any cardiopulmonary symptoms (dyspnea, chest pain or pressure, and/or palpitations). DCE-MRI measurements were evaluated with the presence of any of these cardiopulmonary symptoms at each phase, using the questionnaire from the nearest timepoint to each MRI exam. Additionally, acute phase DCE-MRI measurements were assessed against persistent cardiopulmonary symptoms > 120days post-symptom onset.

Lung CT from the matching study phase were evaluated by attending critical care/pulmonary physicians and radiologists and the final analysis of the images corroborated the presence or absence of ground glass opacities or consolidations. The CT findings were correlated to DCE-MRI quantitative parameters at each study phase. Only CT exams within 60 days of DCE-MRI were included.

In addition to the above analyses, each perfusion parameter was correlated to each clinical metric. The first available exam was used for each patient was used to avoid duplicate inclusion of individuals, and correlation was repeated for the convalescent phase only to assess persistent perfusion abnormalities.

### Statistics

Statistical analysis was performed using GraphPad (Prism 10 v10.2.0, GraphPad Software, Boston, MA). Group-wise comparison used a Mann-Whitney non-parametric test for two groups, Kruskal-Wallis non-parametric test for three groups, or one-way ANOVA for repeated measures. Patients with respiratory comorbidities were excluded from statistical comparison to healthy volunteers. Correlation used Spearman’s rho for non-parametric analysis. Significance was defined as *p* < 0.05.

## Results

### Patient demographics

Patient demographics are provided in Table 1, including vaccine status, COVID variant, and patient risk factors. 10% of the patients had respiratory comorbidities, which may impact pulmonary perfusion. A total of 100 COVID-19 patients were enrolled and imaged in 209 separate DCE-MRI exams. Fifty-three DCE-MRI exams were excluded (19 due to severe respiration image artifacts, 10 due to other image artifacts, 6 due to acquisition error, 7 due to contrast injection failure, and 11 due to other technical issues, summarized in Supplemental Fig. 1). Final analysis was therefore comprised of 84 post-COVID-19 patients and 156 separate DCE-MRI exams. The earliest scan was 8 days post-symptom onset, and the latest follow-up was 1101 days post-symptom onset. Forty-six patients were imaged in the acute phase (median (interquartile range), 32 (26,39) days post-symptom), 50 in the recovery phase (78 (67,86.5) days post-symptom), 47 in the convalescent phase (288 (267.5, 314.5) days post-symptom), 11 in the extended 1 phase (736 (709.5,750.5) days post-symptom), and 2 in the extended 2 phase (1080 (1069.5,1090.5) days post-symptom). Thirty-four patients were imaged only once, 30 were imaged twice, 18 were imaged three times, and 2 were imaged four times across the 5 study phases. In patients where curve fitting failed (Fig. 2B) and PTT and ATT values were non-physiological, these values were removed from data analysis (*n* = 10).

**Table 1:** Baseline characteristics of COVID-19 patients and controls Unless otherwise specified, data are median, with the interquartile range in parentheses, and number (n) and percentage (%) for categorical variable

COVID-19 Patients (*n* = 84)	
Age in years, median (range)	45 (37–55)
Female, n (%)	45 (54)
Race/Ethnicity, n (%)	
White, n (%)	39 (46)
Latino, n (%)	18 (21)
Black, n (%)	19 (23)
Asian, n (%)	8 (10)
Vaccination Status, n (%)	43 (51)
BNT162b2 (Pfizer/BioNTech), n	31
mRNA-1273 (Moderna), n	9
Ad26.COV2.S (Johnson&Johnson), n	3
SARS-CoV-2 Variant	
Pre-Omicron, n (%)	59 (70)
Omicron, n (%)	25 (30)
Comorbidities and Risk Factors	
Hypertension, n (%)	20 (24)
Hyperlipidemia, n (%)	14 (17)
Type 2 Diabetes, n (%)	4 (5)
Coronary Artery Disease, n (%)	1 (1)
Atrial Fibrillation, n (%)	1 (1)
Immunocompromised, n (%)	3 (4)
Asthma, n (%)	6 (7)
COPD , n (%)	1 (1)
Other Chronic Lung Disease, n (%)	1 (1)
History of Venous Thromboembolic Disease, n (%)	4 (5)
Obstructive Sleep Apnea, n (%)	3 (4)
Anemia, n (%)	4 (5)
Hospitalized for COVID-19, n (%)	17 (20)
Disease Severity	
Mild (ambulatory, no oxygen ), n (%)	67 (80)
Moderate (hospitalized with ≤ 6 L/min oxygen by nasal cannula), n (%)	9 (11)
Severe (hospitalized with oxygen > 6 L/min on nonrebreather mask and/or high flow nasal cannula ), n (%)	8 (9)
Healthy Controls (*n* = 10)	
Age in years, median (range)	31 (25–56)
Female, Controls, n (%)	2 (20)

### Perfusion characteristics

**Fig. 3:**
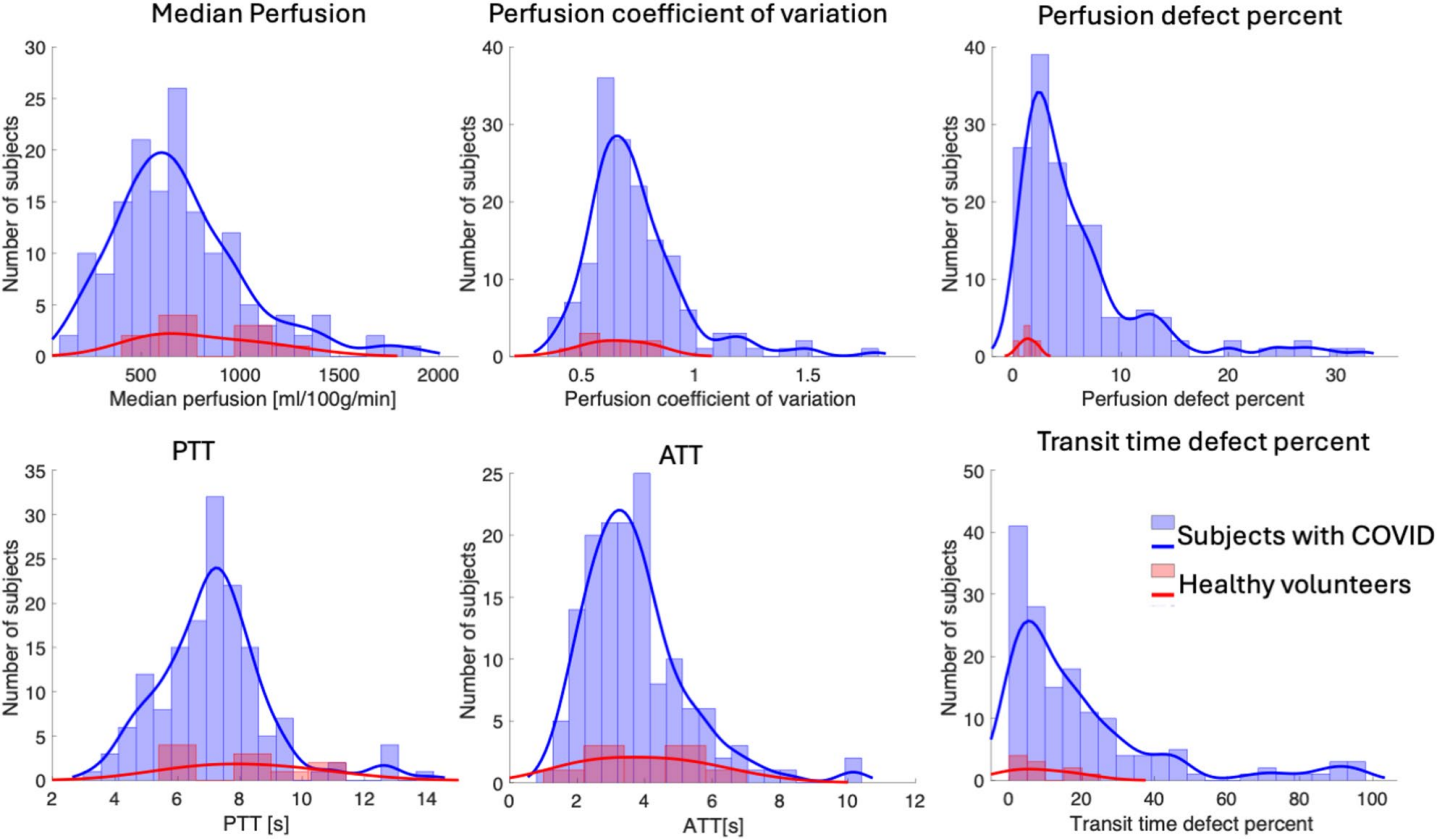
Histogram distributions of pulmonary perfusion parameters: median lung perfusion, perfusion coefficient of variation, perfusion defect percent, pulmonary transit time (PTT), arterial transit time (ATT), and transit time defect percent. Histograms are compared between patients post-COVID-19 from all study phases (blue) and healthy volunteers (red). Both raw histogram data and ‘kernel’ distribution fits are plotted. The 156 exams in patients post-COVID-19 were divided in 20 bins, and the 10 healthy volunteer exams were divided in 5 bins

Figure 3 provides histograms distributions of perfusion quantitative parameters in patients post-COVID-19, compared to healthy volunteers. The 156 exams in patients post-COVID-19 were divided into 20 bins for histogram visualization, and the 10 healthy volunteers were divided in 5 bins, each was fit with a ‘kernel’ estimate of the distribution. Histograms of median perfusion, PTT, and ATT were similar between patients post-COVID-19 and healthy volunteers. Abnormally heterogeneous perfusion (i.e., high coefficient of variation) and perfusion defect percent were more prominent in patients with COVID-19 compared to healthy volunteers and transit time defects were elevated. We identified 5 patients with perfusion defects by visual inspection. Perfusion parameters correlated significantly with age (Supplemental Table 1), and women had statistically higher perfusion than men (*p* < 0.0001).

**Fig. 4:**
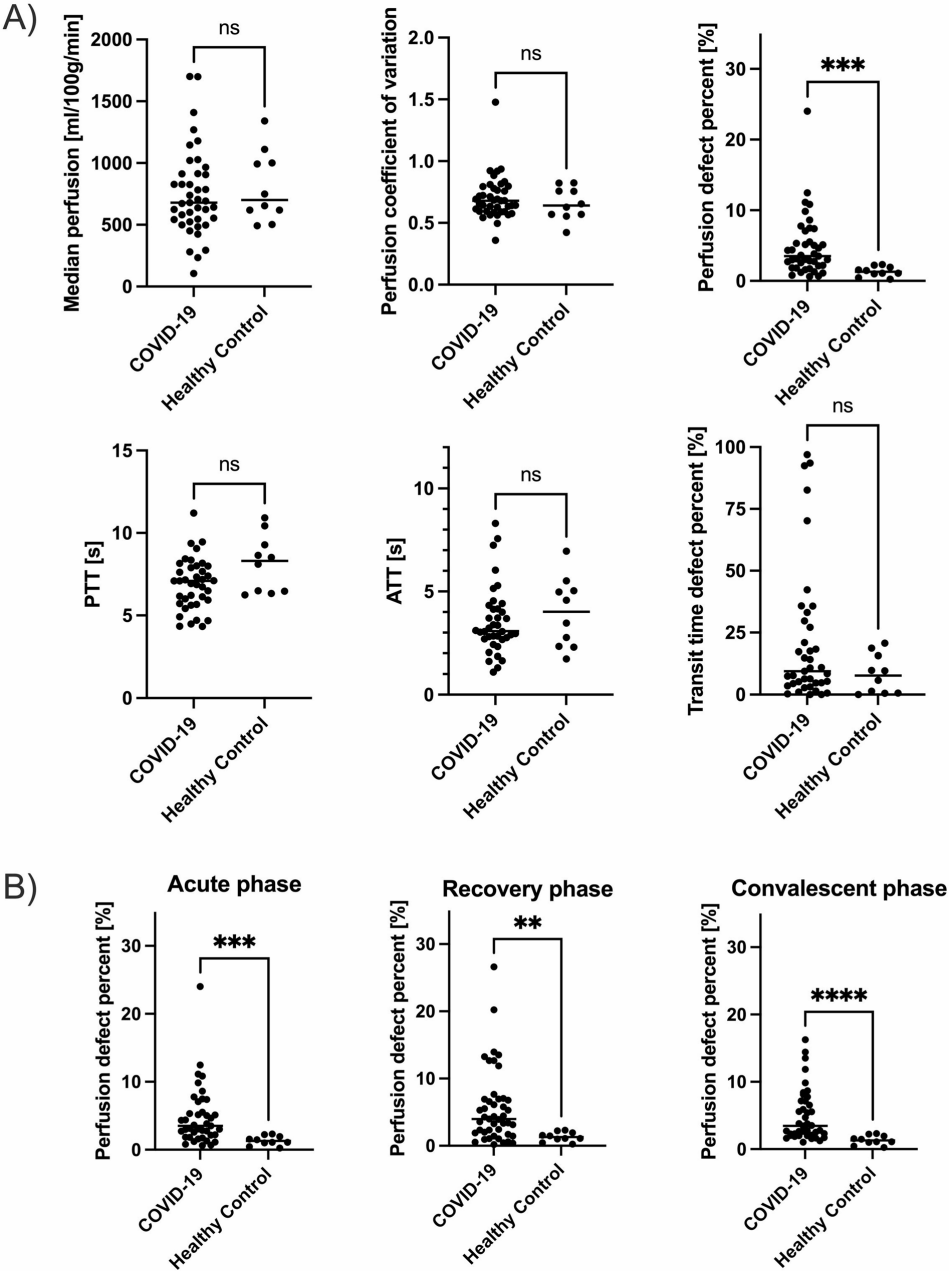
Comparison of patients post-COVID-19 with healthy controls, excluding patients with respiratory comorbidities. **A** Acute phase perfusion parameters were similar between patients with COVID-19 (*n* = 41) and healthy controls (*n* = 10), except for perfusion defect percent. **B** Significantly higher perfusion defect percent in patients with COVID-19 is sustained through later study phases (recovery *n* = 45, convalescent *n* = 41). **p < 0.01, ***p < 0.001, ****p < 0.0001 by Mann-Whitney test

Figure 4 compares perfusion parameters in patients with COVID-19 to healthy volunteers. Patients with respiratory comorbidities were excluded from this comparison in order to assess the impact of COVID-19 infection without confounding underlying disease. Higher perfusion defect percent was observed in patients post-COVID-19 infection compared to healthy volunteers, and statistical significance was maintained across study phases (*p* < 0.01 for all phases, Fig. 4B). The perfusion parameters were similar across study phases (Supplemental Fig. 2, p = ns by repeated measures ANOVA for all comparisons), although one patient with COPD had notably high perfusion defect percent across phases.

### Association with disease severity

**Fig. 5:**
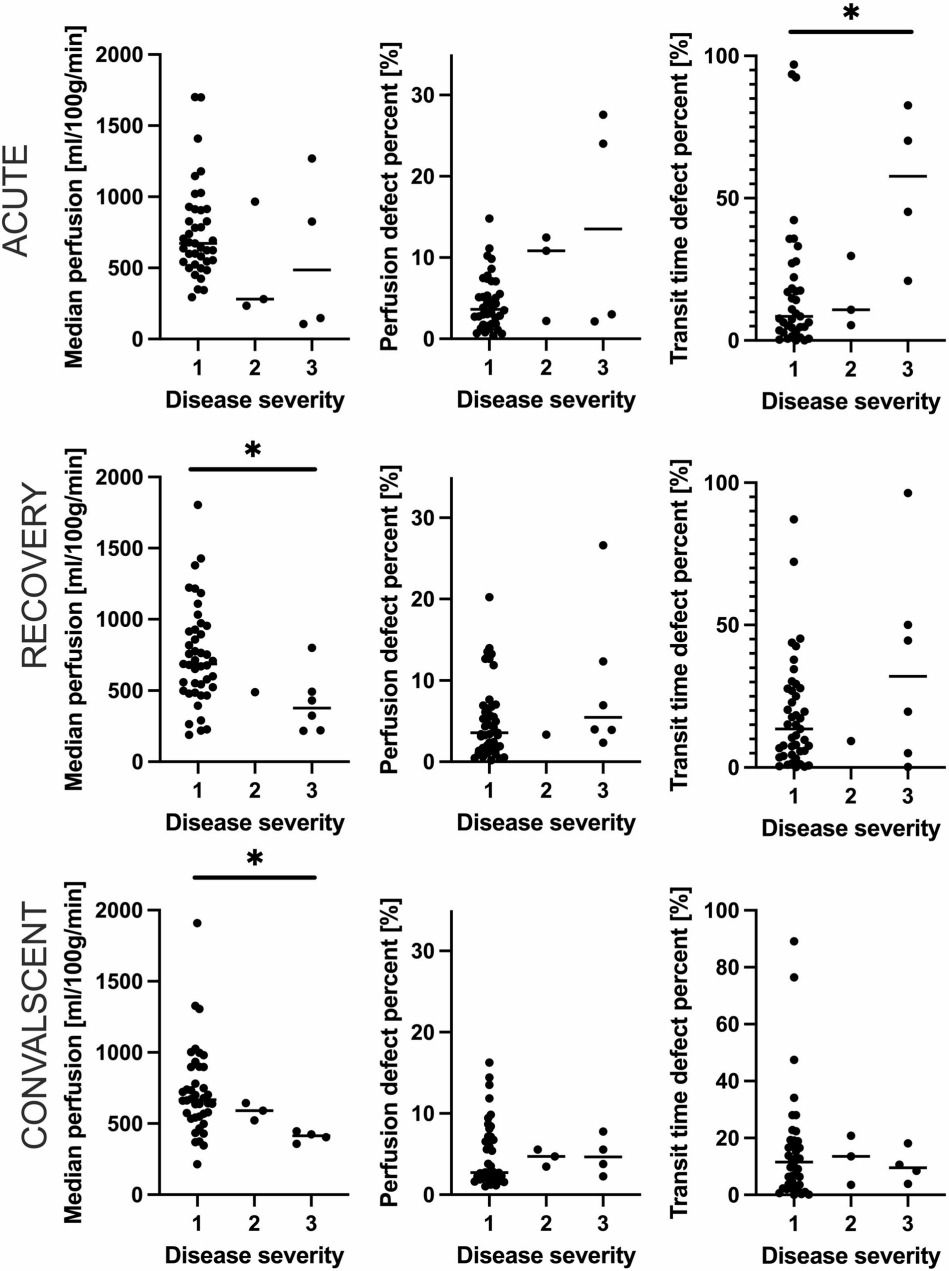
Median perfusion, perfusion defect percent, and arterial transit time (ATT) plotted against disease severity scores (1 = mild, 2 = moderate, 3 = severe) for the acute (*n* = 46), recovery (*n* = 50), and convalescent (*n* = 47) study phases. Trends between disease severity and perfusion measurements were observed, with those that reached statistical significance indicated on plots. *p < 0.05 by Kruskal-Wallis test

Figure 5 shows median perfusion, perfusion defect percent, and transit time defect percent plotted against disease severity for three study phases. Median perfusion was lower during recovery and convalescent phases in patients with more severe disease (*p* = 0.04 and *p* = 0.02, respectively, by Kruskal-Wallis test). Spearman’s correlation also illustrated a significant correlation between disease severity and low perfusion in the convalescent phase (Supplemental Table 1). In the acute phase, the transit time defect percent was higher in patients with more severe disease (*p* = 0.04). In all three phases, there was a trend toward higher defect percent in patients with more severe disease. Relatively few subjects had disease severity score > 1 in our study, which limits comparison.

### Association with pulmonary function tests

**Fig. 6:**
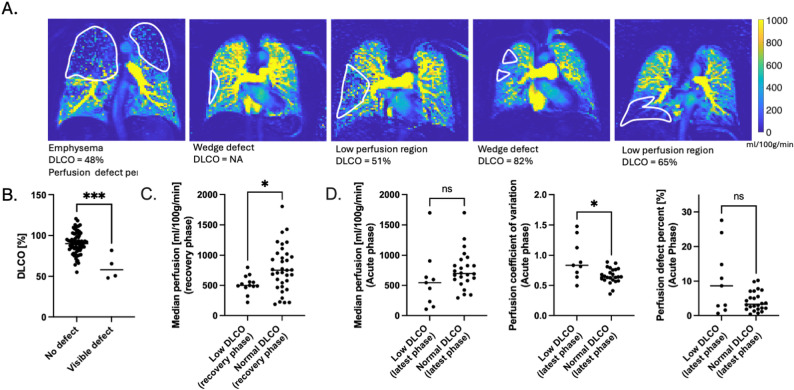
Comparison of perfusion parameters and DLCO. **A** Perfusion maps in the five post-COVID-19 patients visually identified to have regional perfusion abnormalities (outlined in white). **B** The 4 patients with visible regional perfusion defects and PFT data available had lower DLCO compared to the last available DLCO measurement from other patients (*n* = 69). **C** In the recovery phase, median perfusion is lower in patients with low DLCO (*n* = 13 low DLCO vs. *n* = 35 normal DLCO), but this relationship resolves in the convalescent phase. **D** Low median perfusion, high coefficient of variation (i.e., high heterogeneity), high perfusion defect percent in the acute phase are predictive of low DLCO later (*n* = 8 low DLCO, *n* = 24 normal DLCO). Patients are grouped to avoid repeated inclusions of patients with multiple exams. *p < 0.05, ***p < 0.001 by Mann-Whitney test

Figure 6 compares perfusion findings with measured DLCO. PFTs were performed within 60 days of DCE-MRI for 120 exams (median 2 days, minimum 0 days, maximum 60 days), including 57 cases where MRI and PFTs were acquired within 1 day. The five patients with visible perfusion defects (Fig. 6A) were observed to have lower DLCO than the other patients (*p* = 0.0009, Fig. 6B). In patients where DLCO was measured more than once, each patient’s first available timepoint was considered for this statistical comparison (4 DLCO measurements in patients with visible defect vs. 69 measurements in patients with uniform perfusion appearance). Of these patients, one had COPD, causing the perfusion defect, and none of the other patients had past medical history that would contribute to the development of lung perfusion abnormalities. Three of the five patients with a visible perfusion defect had subsequent exams with normal perfusion. Anecdotally, DLCO was lowest during the timeframe with a visible defect. For example, in two patients, DLCO was low when a perfusion defect was visible (65% and 50%), and subsequently increased when the defect resolved (91% and 77%, respectively). In another patient, the DLCO was reduced from 96% (no defect) to 82% (defect visible) later. These examples suggest that some perfusion defects may be transient in nature, which also impacts lung function.

PFT measurements and perfusion parameters were compared in only the recovery and convalescent phases due to PFT data availability. Lower median perfusion was found in patients with low DLCO in the recovery phase (Fig. 6C, *p* = 0.02), and this relationship disappeared in the convalescent phase (*p* = 0.95). Spearman’s correlation analysis showed significant relationships with FEV1/FVC but not DLCO across all subjects and study phases, and an inverse correlation between some perfusion parameters and DLCO during the convalescent phase (Supplemental Table 1).

Abnormal perfusion metrics at the acute study phase was also associated with low DLCO later (Fig. 6D). The time between acute phase perfusion measurement and final study PFT was median 252 days, range 41-1070 days. Patients with heterogeneous perfusion (i.e., higher coefficient of variation) during the acute study phase were more likely to have low DLCO at their final PFT measurement (*p* = 0.01). There were similar trends for patients with low perfusion and high perfusion defect percent.

### Association with cardiopulmonary symptoms

**Fig. 7:**
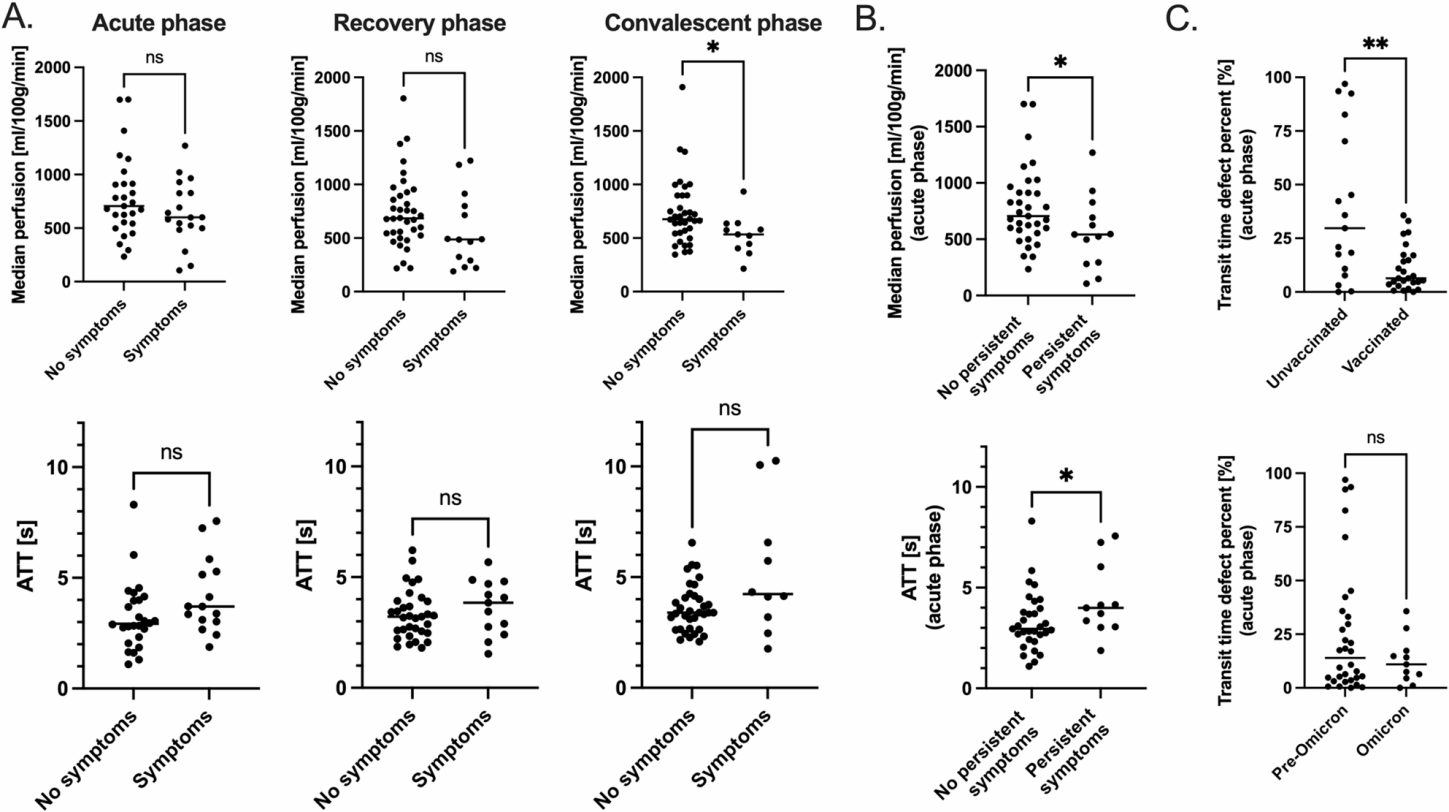
Relationship between perfusion measurements and cardiorespiratory symptoms. **A** Relationship across the acute phase (*n* = 27 without symptoms, *n* = 18 with symptoms, *n* = 1 unknown), recovery phase (*n* = 36 without symptoms, *n* = 14 with symptoms), and convalescent phase (*n* = 36 without symptoms, *n* = 11 with symptoms) phases. **B** Perfusion measurements in the acute phase were predictive of persistent symptoms in the convalescent phase (*n* = 33 without persistent symptoms, *n* = 13 with persistent symptoms). **C** Transit time defect percent in the acute phase versus vaccination status (*n* = 18 vaccinated and *n* = 27 vaccinated) and variant (*n* = 33 pre-omicron and *n* = 12 omicron). *p < 0.05, **p < 0.01 by Mann-Whitney test

Figure 7 compares median perfusion and ATT with concurrent symptom reporting and in patients with persistent symptoms. Cardiopulmonary symptoms evaluated at each timepoint were related to measured perfusion parameters (Fig. 7A). Across all phases, median perfusion was lower and ATT was higher in patients with symptoms. This trend reached statistical significance (*p* = 0.01) for median perfusion measured in the convalescent phase. In patients with persistent symptoms (> 120 days post-infection), we found that high ATT or low perfusion in the acute phase were both associated with persistent symptoms (Fig. 7B, *p* = 0.04 and *p* = 0.04). High ATT indicates slow delivery of blood from the main pulmonary artery to the lung, reflecting some pulmonary vascular dysfunction. Spearman’s correlation also showed significant relationship between low perfusion and symptoms in the convalescent phase (Supplemental Table 1).

### COVID-19 variant and vaccination status

The transit time defect percent was lower in the acute phase in vaccinated individuals compared to non-vaccinated (*p* = 0.0047), and trended lower in patients with pre-omicron variants (Fig. 7C). The transit time defect percent indicates regions of slow blood delivery, and our results indicate that unvaccinated individuals had more regional deficits. No other significant differences in perfusion parameters in the acute phase were identified between pre-omicron and omicron variants or with vaccine status.

### Association with other imaging findings

CT imaging was performed within 60 days for 150 MRI exams (median 0 days, minimum 0 days, maximum 42 days), including 112 cases where MRI and CT exams were within 1 day. Patients with ground glass opacities or consolidation were compared to those without these findings. In the convalescent phase, patients with ground glass opacities had significantly lower median perfusion (*p* = 0.004), indicating persistence of both structural and functional abnormalities in some patients. However, there were no other associations between CT findings and perfusion parameters in the acute or recovery phases, indicating that microvascular disease assessed using pulmonary perfusion measurements is not always reflected in the morphological changes associated with ground glass or consolidation.

## Discussion

We report on longitudinal DCE-MRI measurements of pulmonary perfusion in 84 patients with a total of 156 exams following COVID-19 infection. To our knowledge, this is the largest study of DCE-MRI in COVID-19 infection. Patients were enrolled starting approximately 1-week post-infection, and the last follow-up was more than 3 years post-infection. We used pharmacokinetic modelling and image processing to generate several perfusion metrics of interest, and performed an exploratory analysis of the relationship between perfusion parameters and clinical metrics. Additionally, visible perfusion defects, indicative of vascular obstruction or vasoconstriction, were observed in 5 individuals.

The associative findings across study phases are summarized in Table 2. One key finding is the importance of the regional measurements. We found that regional assessments of perfusion defects and perfusion heterogeneity were important in detecting differences between the acute phase and persistent clinical abnormalities. This emphasizes the need for regional imaging to detect functional abnormalities, especially in subclinical disease. Another key finding is the relationship between impaired perfusion parameters and reduced DLCO. These findings align well with other published data on dual-energy CT and hyperpolarized xenon MRI [27, 28]. It is of clinical relevance that we found an association between vaccination and reduced perfusion abnormalities, indicating that vaccination is mitigating subclinical disease in this mostly ambulatory population.

**Table 2:** Summary of findings for each perfusion metric across three study phases

	Acute	Recovery	Convalescent
Median perfusion (low = abnormal)	• Associated with persistent symptoms	• Lower with more severe disease	• Lower with more severe disease
		• Lower with low DLCO	• Correlation with disease severity, ground glass opacity, and symptoms
Perfusion coefficient of variation (high = abnormal)	• Associated with low DLCO later		
Perfusion defect percent (high = abnormal)	• Higher post-COVID-19 vs. healthy	• Higher post-COVID-19 vs. healthy	• Higher post-COVID-19 vs. healthy
PTT (high = abnormal)			
ATT (high = abnormal)	• Associated with persistent symptoms		• Higher with symptoms
Transit time defect percent (high = abnormal)	• Higher in patients with more severe disease		
	• Lower in vaccinated individuals		

COVID-19 infection is known to cause microvascular disruptions in the lung, including microemboli and vasoconstriction, and our quantitative perfusion parameters are indicative of microvascular dysfunction. Our results showed low median perfusion was associated with persistent symptoms and perfusion remained low in convalescent phase for patients with severe disease; we found high perfusion defect percent compared to healthy controls; and we found long transit times were present in patients with symptoms. These findings support the presence of microvascular perfusion abnormalities in patients with symptomatic or severe disease even months after COVID-19. The underlying source of these disruptions, such as vascular remodeling or residual emboli, are unclear [4, 29]. Notably, the majority of our patients were ambulatory and not severely ill suggesting that microvascular abnormalities are an underappreciated consequence of this infection. Our findings also indicate that DCE-MRI measurements of lung perfusion were independent of CT structural observations, except in the convalescent phase, indicating that pulmonary perfusion MRI may be able to provide additional complementary information about pulmonary physiology that is not attainable through other imaging means. Prior studies using dual-energy CT found perfusion defects in proximity to parenchymal lesions on initial CT scans [30], which differs from our findings. This discrepancy may be due to the acute disease stage during their CT measurement (patients with positive reverse transcription polymerase chain reaction). The authors also found ground glass opacities are present in the patients with residual perfusion defects during follow-up scans > 80 days after the initial scan, which corresponds to our MRI findings.

The link between abnormal perfusion in the acute disease phase, and later findings of low DLCO in the same patient, offers a glimpse into the predictive nature of this measurement, indicating that early perfusion abnormalities may relate to long term detrimental effects on lung function. We primarily investigated DLCO in PFT analysis because it is expected that regional perfusion deficits are most likely to impair the diffusion of gas into the blood, the correlative findings with FEV1/FVC and inverse correlation with DLCO at convalescence were unexpected.

Other publications investigating DCE in COVID-19 infection have used “semi-quantitative” analysis of the contrast agent wash-in rate, wash-out rate, and time-to-peak. Yu et al. found that late contrast bolus arrival was correlated with post-COVID-19 dyspnea in males [10] using a metric akin to our transit time defect in their study. Similarly, Zhou et al. found that wash-in was slower and the contrast peak wider in patients post-COVID-19 for a region of interest [9]. Saunders et al. used pharmacokinetic modelling for quantitative perfusion measurements with DCE-MRI in 25 exams across 8 patients and measured a non-significant increase in perfusion between repeat visits [15]. Other MRI methods including non-contrast “PREFUL” [9, 10, 31, 32] and dissolved phase imaging of hyperpolarized xenon [15, 28] have demonstrated discrepancies in V/Q mismatch and reduced gas transfer in patients post-COVID-19 and with long COVID-19 symptoms. Our study bolsters these prior studies of pulmonary perfusion following COVID-19 by finding similar abnormalities in a larger patient population and demonstrating the association between quantitative perfusion metrics and clinical findings.

We chose to pursue fully quantitative perfusion analysis from DCE-MRI via pharmacokinetic modelling. We selected the two-compartment exchange model, which is appropriate for highly permeable tissues [11]. We used an open-source and validated software package, ROCKETSHIP, for quantitative analysis [23, 33], and assumed a constant pulmonary T1. Using this model, we measured a median pulmonary perfusion in the lung of 693 ± 333 ml/100 g/min (averaged across all patients). A recent meta-analysis of MRI and CT contrast enhanced perfusion reported pulmonary perfusion values in the range of 100-700 ml/100 g/min for healthy volunteers [8]. Our measured values were on the higher end of this range, which may be due to the use of a two-compartment exchange model which includes an estimation of permeability, whereas other models used in the lung do not. Additionally, we are using a novel 0.55T MRI system, for which the signal response of gadolinium in the lung has not been previously characterized or the contribution of major vessels which were not excluded in segmentation. The pharmacokinetic model also provides estimates of other parameters (e.g., pulmonary blood volume and permeability), which warrant additional investigation in future studies. Women had higher perfusion (median 707 ml/100 g/min) compared to men (median 542 ml/100 g/min) in our study, which matches previous findings of perfusion in the brain and heart [34, 35].

Our study used a novel ramped-down 0.55T MRI system with high-performance hardware. We have previously demonstrated the superior lung imaging capabilities of this system, by virtue of the improved field homogeneity and reduced off-resonance artifacts caused by air-tissue interfaces [6, 22, 36, 37]. At this field strength, we also expect reduced T2* effects from gadolinium injection in the lungs. Contemporary 0.55T MRI systems have been used to image lung morphology, including in COVID-19 [21, 38, 39]. However, for the present study, we opted to compare our measurements of quantitative perfusion with gold-standard morphological assessment via chest CT. One advantage of this 0.55T MRI system is the lower cost compared to conventional 1.5T and 3T MRI, which may improve the accessibility and widespread use of such lung MRI approaches.

Our study has several limitations. First, our acquisition was limited by a low spatial resolution and long breath-hold. While our breath-hold length was similar to previous pulmonary DCE studies in COVID-19 [10], respiratory artifacts were still observed in a number of participants. Several scans were also excluded due to respiration artifacts or technical issues using our investigational MRI system. It possible that patients who could not hold their breath had pathology causing respiratory dyskinesis, and therefore our final results may be weakened by excluding some patients with this manifestation of disease. Second, our cohort size was limited and although our study design enabled the longitudinal monitoring of patients post-COVID-19, many patients were scanned at only 1 or 2 timepoints, which limits data analysis and interpretation. We chose non-parametric tests for group-wise comparisons based on our data, and the reported p-values must be taken in the context of the study. Additionally, we only included 10 healthy controls for these contrast-enhanced scans, and healthy controls were on-average 14 years younger that patients and predominantly male. Third, PFT data was unavailable for most patients in the acute phase as our PFT laboratory did not operate in the height of the pandemic. Fourth, we used a gadolinium-based contrast agent to measure perfusion, which may not be suitable in patients with renal dysfunction. An endogenous contrast agent approach, such as arterial spin labelling [14], would be preferred in the future. Fifth, we do not have control measurements pre-infection for these patients to isolate the effects of COVID-19 versus pre-existing abnormalities. Finally, there is potential bias in patient symptom self-reporting.

In conclusion, quantitative pulmonary perfusion by MRI showed measurable differences from healthy volunteers, across longitudinal timepoints, and associated with clinical metrics and persistent symptoms. Our results indicate that DCE-MRI is sensitive to pulmonary microvascular perfusion abnormalities found months after COVID-19 which may contribute to the persistence of pulmonary symptoms.

## Supplementary Information

Supplementary Material 1.

Supplementary Material 2.

Supplementary Material 3.

## Data Availability

Data are available from the corresponding author on reasonable request.
