# Supplementary material for: A longitudinal study of quantitative pulmonary dynamic contrast enhanced MRI following COVID-19 infection
Source: Respir Res. 2026 Mar 26;27:221. doi: 10.1186/s12931-026-03582-w (PMC13244964; doi:10.1186/s12931-026-03582-w)
Supplement: Supplementary file 1 — Supplementary Material 1. [file 12931_2026_3582_MOESM1_ESM.docx]

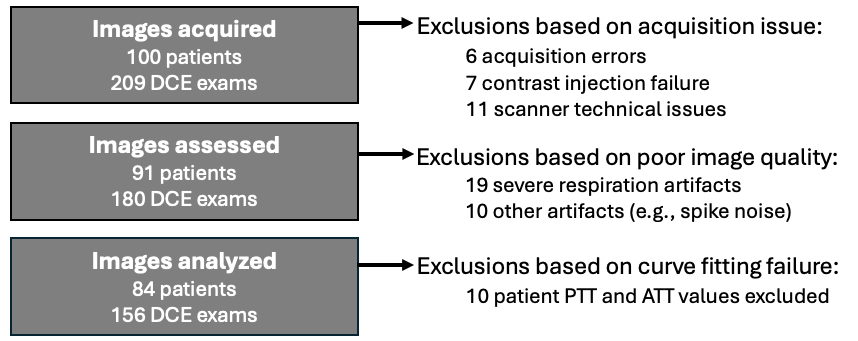


Supplemental Figure 1: Summary of data exclusion based on image acquisition, image quality assessment, and analysis failures.


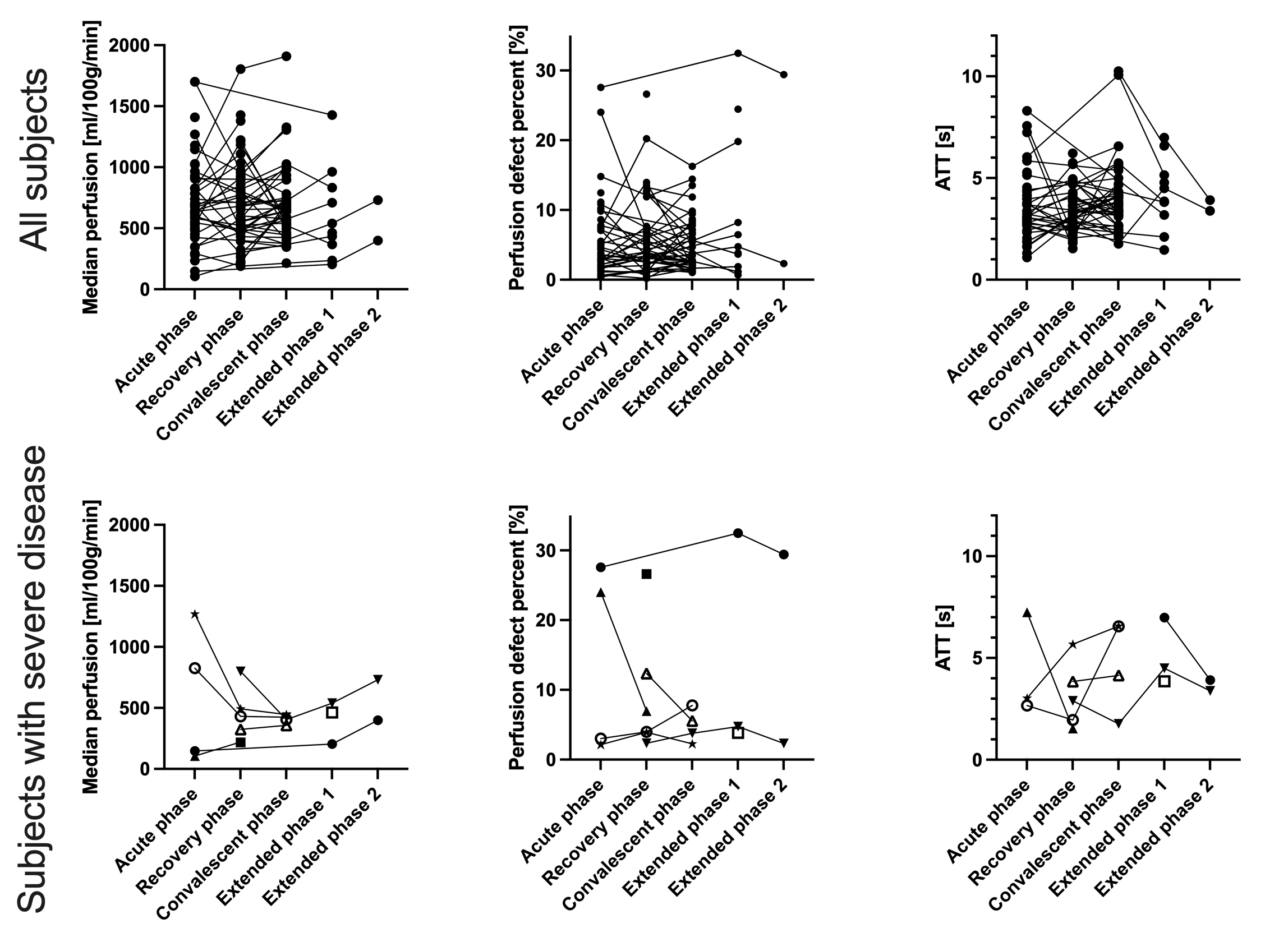


Supplemental Figure 2: Median perfusion, perfusion defect percent, and arterial transit time (ATT) plotted across study phase for all patients post-COVID-19 (top row, n = 46 acute, n = 50 recovery, n = 47 convalescent, n = 11 extended1 , n = 2 extended 2) and only those identified to have severe disease (bottom row, n = 4 acute, n = 6 recovery, n = 4 convalescent, n = 3 extended 1, n = 2 extended 2). One patient with emphysema had high perfusion defect percent across phases. No statistically significant differences across phases were measured (repeated measures one-way ANOVA).


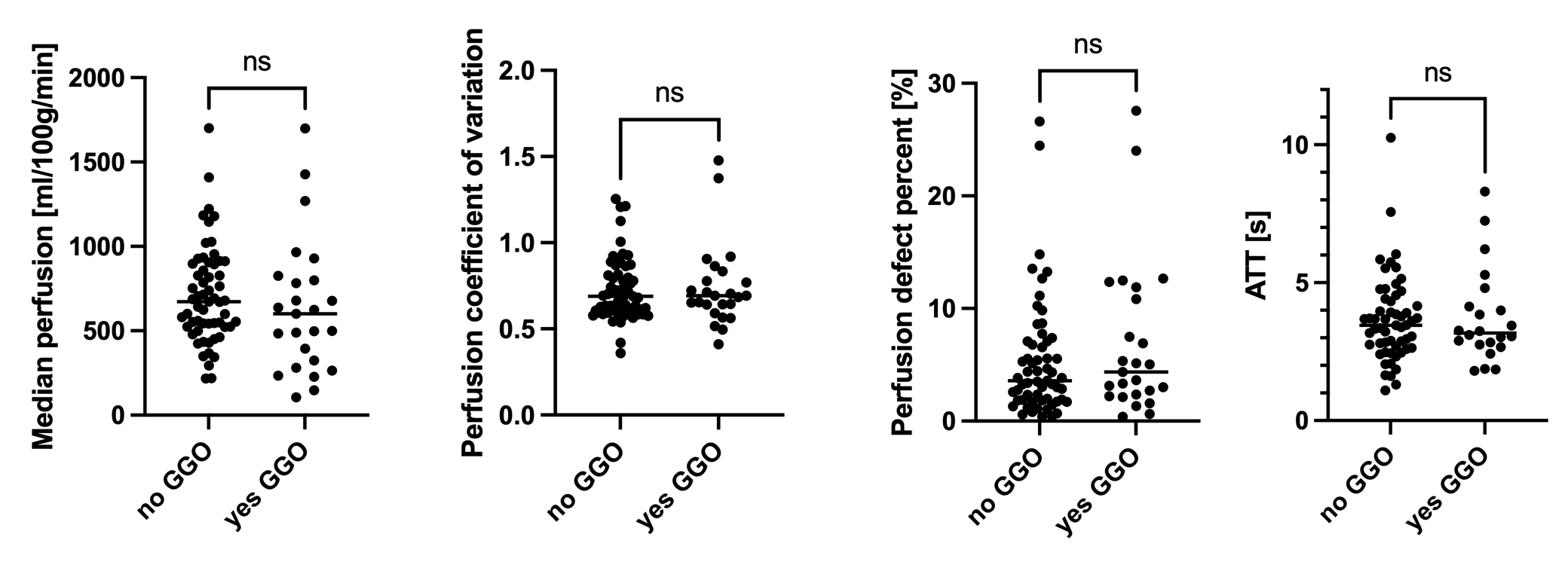
Supplemental Figure 3: No relationship between perfusion measurements and CT findings of ground glass or consolidation was observed (n = 59 without GGO, n = 25 with GGO). The first available measurement from each patient was included.

Supplemental Video 1: Dynamic contrast enhanced pulmonary MRI across 6 slices. Patient with wedge-shaped perfusion defect, corresponding to Figure 1, is shown as an example.
